# Reflections on the responsible conduct of cancer research

**DOI:** 10.1186/1475-2867-10-5

**Published:** 2010-03-02

**Authors:** Mark A Brown, Richard J Ablin, Denys N Wheatley

**Affiliations:** 1 Colorado State University, 801 Oval Drive, Fort Collins, CO 80523-1052, USA; 2 University of Arizona College of Medicine, Arizona Cancer Center, BIO5 Institute, Center on Injury Mechanisms & Related Responses, 1501 N. Campbell Avenue, Tucson, AZ 85724-5221, USA; 3 Cancer Cell International, BioMed Central Ltd. Floor 6, 236 Gray's Inn Road London WC1X 8HL, UK; 4 BioMedES, Leggat House, Keithhall, Inverurie, Aberdeen AB51 0LX, UK

## Abstract

Most cancer researchers regularly practice the responsible conduct of research (RCR) without consciously considering it. As professional scientists, we simply do what we are trained to do. However, as we train a new generation of cancer researchers in our laboratories, we must be vigilant against undue complacency. In an age when misconduct in research is receiving more media attention than ever before, we should periodically take a moment of pause and reflect upon the meaning and practice of responsibly conducting research. Rather than meeting minimum standards in a compliance-driven manner, we should practice forethought and periodically consider how we can improve. We, as leaders in cancer research, must then push our peers to do the same. By embedding RCR into the culture of cancer research through a multilayer approach, including regular assessment at the levels of individual research groups, departmentally, and institutionally, we will become a model discipline in the responsible conduct of research.

## Editorial

As one of the leading causes of death worldwide, cancer is widely regarded as the bane of humanity. Driven by the fear associated with this surreptitious killer, public support for cancer research has grown dramatically throughout the last 50 years. A century ago, little was known about this disease. Today, few lives have been untouched by the devastating impacts of cancer. Increasing public support for cancer research has elicited well warranted trepidation about the way it is conducted. Public funds support an increasing share of all cancer research throughout the world. Therefore, cancer researchers spend a growing portion of their time employed, indirectly, by the public. As public servants, however indirect the actual line of accountability may be, it is incumbent upon cancer researchers to ensure the responsible conduct of their research. This should be done in a way that makes their work more transparent and understandable through communications that are accessible to the general public. With the advent of public access to the primary literature documenting cancer research, public awareness has grown regarding the rare but destructive spectre of research misconduct. In response, many public funding agencies have endorsed new policies regarding the *responsible conduct of research *(RCR). These policies are intended to ensure a culture of ethical practices in research, to safeguard the integrity of the research findings upon which we rely for therapeutic advances in health care, and alleviate public reservations regarding the potential misuse of public funds. As pioneers at the frontier of cancer research, we would be well advised to take a moment of pause to consider the relevance of RCR in cancer research.

Across disciplines, responsible conduct in research is simply the practice and reporting of sound, ethical science. It is paramount that biomedical researchers conduct their work truthfully, professionally, and impartially using the best ethical practices. RCR is particularly critical in the sensitive field of cancer research because it touches the lives of so many. As cancer researchers, we are responsible not only for the timely progress of 'fundamental studies on cells prior to, during and after transformation' [1] but also for the training of the next generation of cancer researchers to continue, responsibly, that progress. Thus, as we reflect upon our own practices and how they contribute to responsible cancer research, we should also consider how we impart the values and standards that drive those practices to our students, post-docs, and other research personnel.

To establish a common tool to guide the reflection upon our own conduct of research, the *Office of Research Integrity *in the United States Department of Health and Human Services (HHS) has identified and published core factors of RCR [2]. Curator of the world's largest biomedical research consortium, HHS has a vested interest in helping to guide and facilitate RCR in the United States and throughout the world. The cores of RCR endorsed by HHS are: *1) research misconduct; 2) conflicts of interest; 3) data management practices; 4) mentor and trainee responsibilities; 5) collaborative research; 6) authorship and publication; 7) peer review; 8) protection of human rights and welfare of laboratory animals; 9) societal expectations of scientists, environmental and societal impacts of scientific research, and ethical considerations in biomedical research*. The meaning and value of each of these cores should be self evident to any biomedical researcher. Each can be more thoroughly reviewed and considered in the introduction to RCR published by HHS [2]. As cancer researchers, we regularly practice the cores of RCR without consciously considering them. We simply do what we are trained to do. However commonplace these cores may be in our daily routine, we must be vigilant against undue complacency. Rather than meeting minimum standards in a compliance-driven manner, we should practice forethought and periodically reflect upon how we can improve. We, as leaders in cancer research, must then push our peers to do the same. By embedding RCR into the culture of cancer research through a multilayer approach, including regular assessment at the levels of individual research groups, departmentally, and institutionally, we will become a model discipline in the responsible conduct of research.

## Competing interests

The authors declare that they have no competing interests.

## Authors' contributions

MAB was responsible for the original draft of this manuscript. RJA and DNW provided critical review and revision of the manuscript. All authors read and approved the final manuscript.

## Authors' information

Dr Denys N Wheatley is Director of BioMed Educational Services (BioMedES).
